# A Case of Severe Acute Respiratory Distress Syndrome Secondary to Atypical Amniotic Fluid Embolism

**DOI:** 10.7759/cureus.28808

**Published:** 2022-09-05

**Authors:** Waqas Rasheed, Saria Tasnim, Anass Dweik, Muhammad S Anil, Muhammad Ali Anees

**Affiliations:** 1 Internal Medicine, Texas Tech University Health Sciences Center, Amarillo, USA; 2 Internal Medicine, Texas Tech University Health Sciences Center, Texas, USA; 3 Internal Medicine, Beaumont Hospital, Dearborn, USA

**Keywords:** pregnancy complication, acute onset severe hypoxic respiratory failure, atypical amniotic fluid embolism, acute respiratory distress syndrome [ards], afe (amniotic fluid embolism)

## Abstract

Acute respiratory distress syndrome (ARDS) is a noncardiogenic pulmonary edema that leads to acute respiratory distress. It remains one of the major diagnoses requiring ICU admission and mechanical ventilation. We present a case of a 25-year-old gravida 3 para 2 female who was admitted for uncomplicated 38-week pregnancy and delivered a healthy male infant but developed acute onset dyspnea six hours after vaginal delivery. She required mechanical ventilation four hours after the onset of respiratory distress and had to be transferred to a higher level facility for extracorporeal membrane oxygenation (ECMO) within 24 hours of the symptom onset. She was diagnosed with severe ARDS. Even though she missed the other typical feature of amniotic fluid embolism, atypical amniotic fluid embolism remained the most likely explanation for her symptoms after the other causes of ARDS were excluded.

## Introduction

Acute respiratory distress syndrome (ARDS) is a noncardiogenic pulmonary edema secondary to inflammation-related lung injury from a variety of causes. It is diagnosed clinically as it is characterized by acute onset bilateral pulmonary infiltrates associated with worsening dyspnea once cardiogenic pulmonary edema and alternate causes of acute hypoxic respiratory failure are ruled out. Pulmonary infiltrates in ARDS are caused by diffuse inflammatory lung injury from a variety of insults including sepsis, pneumonia, aspiration, toxin inhalation, blood transfusion reaction, pancreatitis, amniotic fluid embolism, and many other causes [1]. Amniotic fluid embolism (AFE) is a rare cause of ARDS in the immediate post-partum period and is one of the most severe complications of pregnancy. It is seen in about one out of 40,000 deliveries, and mortality ranges between 20% and 60% [2]. We present a patient who developed hypoxic respiratory failure six hours after delivery and required endotracheal intubation and mechanical ventilation within four hours of symptom onset. Narrowing down the differential diagnosis, atypical AFE remained the most likely explanation for her symptoms. The purpose of this case presentation is to share an unusual and rare case of ARDS secondary to atypical AFE where many of the clinical features of AFE were missing.

## Case presentation

**Figure 1:**
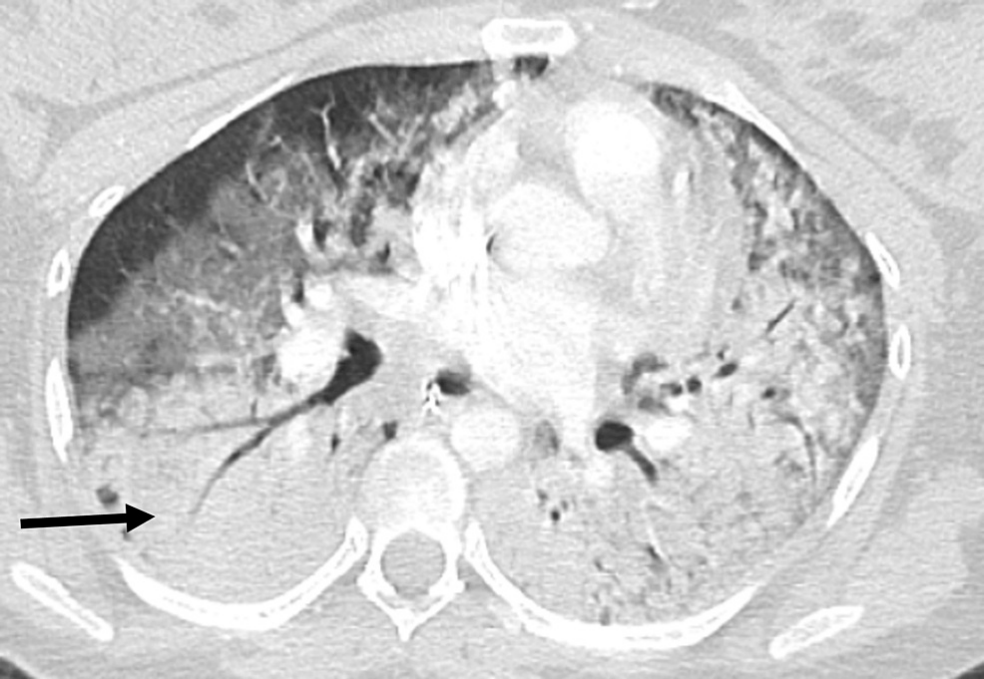
Dense bilateral pulmonary infiltrates on CT scan chest indicating ARDS (as indicated by the arrow) ARDS: Acute respiratory distress syndrome.

A 25-year-old gravida 3 para 2 Hispanic female with anemia of pregnancy and no other medical problems was admitted to the labor and delivery unit at 38 weeks gestation while she was in labor. She followed up with her obstetrician regularly throughout her pregnancy, and her pregnancy course had been uncomplicated. She did not have any history of complicated pregnancy or delivery in the past, no history of surgeries in the past, did not smoke cigarettes or drink alcohol, and did not use any illegal drugs. Her home medications included ferrous sulfate 325 mg daily and prenatal vitamins. The vital signs at presentation were pulse of 89 bpm, blood pressure of 130/85 mmHg, respiratory rate of 18/min, temperature of 98.6°F, and oxygen saturation of 97% on room air. The physical examination was normal, including normal respiratory and cardiovascular examination. The pelvic examination done by the obstetrician was normal for gestational age, and the fetal heart rate was also normal with moderate variability and no decelerations. Her only symptoms were related to her labor, and she denied any recent fever or any respiratory or cardiovascular symptoms. Laboratory workup showed white blood cell (WBC) count of 15.9/mcL (4-10.6/mcL) with 90% neutrophils and hemoglobin (Hb) of 8.8 mg/dL (12-16 mg/dL) with a low mean corpuscular volume (MCV) and mean corpuscular hemoglobin concentration (MCHC) indicative of microcytic and hypochromic anemia, consistent with her previously diagnosed anemia of pregnancy. A healthy male infant was delivered vaginally within one hour of admission, without requiring any instrumentation or surgical procedure, a complete placenta was removed, and a second-degree laceration was successfully repaired. No postdelivery bleeding or any other complications were noted. The patient started complaining of dyspnea six hours after vaginal delivery and developed tachycardia and tachypnea. There was no chest pain, loss of consciousness, nausea, or vomiting, and she was not given any new medicines/food or blood transfusion before/during her symptoms started. Vital signs were as follows: pulse 142 bpm, blood pressure 140/78 mmHg, and temperature 97°F. CT pulmonary angiography (CTA) was performed to rule out pulmonary embolism (PE), which was negative for PE but showed dense bilateral opacities (Figure 1).

CT scan of the abdomen and pelvis with contrast only showed an enlarged uterus, and no other abnormalities were observed. Bedside transthoracic echocardiogram (TTE) showed left ventricular ejection fraction (EF) > 75%, without diastolic dysfunction or hemodynamically significant valvular abnormalities, and there were no vegetations. The patient was transferred to ICU as her oxygen saturation started dropping. She was started on oxygen, initially, a Venturi mask, which was later switched to a nonrebreather mask. However, hypoxia was not corrected, and tachypnea and tachycardia kept getting worse, ultimately requiring endotracheal intubation and mechanical ventilation within four hours of symptom onset. Arterial blood gases (ABGs) showed hypoxic respiratory failure, and the initial PaO_2_/FiO_2_ ratio was 95 (PaO_2_ 38 mmHg while on Venturi mask with 40% oxygen saturation). The patient remained hemodynamically stable, no pressor support was required, and no cardiac arrhythmias were observed. Her physical examination at this point revealed bilateral crackles; however, the rest of the physical exam including the gynecological exam remained unremarkable, without any evidence of bleeding or infection. Repeat laboratory workup showed worsening leukocytosis with neutrophilic predominance and lymphopenia (Table 1). Blood and sputum cultures were sent, and broad-spectrum antibiotics including piperacillin-tazobactam 4-0.5 g every eight hours and linezolid 600 mg every 12 hours were started while awaiting culture results. The patient was on high ventilator support at this time and was transferred to a higher level of facility for extracorporeal membrane oxygenation (ECMO).

**Table 1:** Laboratory findings

Laboratory workup	0 hours	10 hours	24 hours	Reference range
White blood cells (WBC)	15.9	21.7	26.9	5.9-16.9/mcL
Absolute neutrophil count (ANC)	14.3	19.9	24.6	3.9-13.1/mcL
Absolute lymphocyte count	0.8	0.8	0.7	1-3.6/mcL
Hemoglobin (Hb)	8.8	7.4	7.7	9.5-15.0 g/dL
Platelets	156	207	209	146-429/mcL
Prothrombin time (PT)		10.4	10.3	9.6-12.9 seconds
International normalization ratio (INR)		1	0.99	0.8-1.09
Partial thromboplastin time (PTT)		24.5	55	22.6-35 seconds
Sodium		138	139	130-148 mmol/L
Potassium		3.5	3.6	3.3-5.1 mmol/L
Chloride		108	107	97-109 mmol/L
Bicarbonate		22	25	12-22 mmol/L
Creatinine		0.5	0.6	12-22 mmol/L
Blood urea nitrogen (BUN)		10	10	3-11 mg/dL
Calcium		7.1	7.8	8.2-9.7 mg/dL
Total bilirubin		0.35	0.29	0.1-1.1 mg/dL
Alkaline phosphatase (ALP)		180	168	38-229 units/L
Aspartate transaminase (AST)		32	39	4-32 units/L
Alanine aminotransferase (ALT)		15	18	2-25 units/L
Lactic acid			2.2	0.4-2.0 mmol/L

## Discussion

ARDS was first recognized in the 1960s and described as acute onset hypoxia, dyspnea, and loss of lung compliance after a variety of stimuli, which did not respond to usual respiratory therapy. It was later called adult respiratory distress syndrome due to its similarity to infant respiratory distress syndrome and was finally named ARDS [3]. Normal healthy lungs performed gas exchange as ventilation (V) matches perfusion (Q). At rest, the normal V/Q ratio is approximately equal to 1. This ratio is disturbed in many cardiorespiratory pathologies including ARDS. A normal alveolar-capillary unit consists of capillary endothelium, capillary basement membrane, interstitial space, alveolar epithelium, and alveolar basement membrane. On average, this barrier is only 0.5 µm thick, which allows excellent gas exchange, provided V matches Q. This barrier is disturbed in ARDS. ARDS results from one or more insults that cause diffuse lung injury. The injury results in pro-inflammatory cytokine release including interleukins (IL-1, IL-6, and IL-8) and tumor necrotic factor (TNF), which mediate damage and activation of the endothelium, resulting in increased permeability and release of fluid and proteins into the interstitium, leading to interstitial edema. This protein-rich edema also interferes with surfactant function, leading to an increase in lung compliance. The overall effect is V/Q mismatch leading to impaired gaseous exchange and a decrease in lung compliance causing stiff lungs [1,4]. There are more than 60 etiologies resulting in ARDS, and the list continues to get longer. The most common causes of ARDS include pneumonia, sepsis, and aspiration [5]. Our patient was diagnosed with ARDS using the Berlin definition. A list of differential diagnoses leading to ARDS in our patient with supporting and negating evidence is listed below (Table 2). 

**Table 2:** Suspected causes of ARDS in our patient with supporting and negating evidence ARDS: Acute respiratory distress syndrome; SIRS: Systemic inflammatory response syndrome; DIC: Disseminated intravascular coagulopathy.

Suspected differentials as a cause of ARDS in our patient	Supporting evidence	Negating evidence
Sepsis	Sepsis is the most common cause of ARDS. The repair of a second-degree vaginal tear was recently performed. Fever, tachycardia, tachypnea, and leukocytosis were present. The patient meets the SIRS criteria for sepsis (temperature > 100, HR > 90, RR > 20, WBC > 12,000).	Sudden onset of severe ARDS in a hemodynamically stable patient without definitive sepsis is unlikely secondary to sepsis. The symptoms started within six hours of vaginal tear repair, and the development of sepsis and causing ARDS is unlikely to happen within six hours only.
Infectious pneumonia	Infectious pneumonia is a common cause of ARDS. Dyspnea, tachypnea, fever, and tachycardia were present when the symptoms started. Worsening leukocytosis was observed as the symptoms worsened. Dense bilateral pulmonary infiltrates were present, and pneumonia can have a similar appearance on imaging.	No cough, dyspnea, or fever were present at the time of admission. The symptoms started within six hours of admission and quickly worsened, which is a short time for pneumonia to develop and get worse. Respiratory cultures and respiratory viral PCR panel including COVID-19 were negative, and no definitive respiratory infection was found.
Aspiration pneumonia	Aspiration pneumonia is a common cause of ARDS. Sudden onset of symptoms may be seen with aspiration pneumonitis.	The patient did not have any risk factors for aspiration. No nausea, vomiting, loss of consciousness, or any other aspiration events were observed before the symptoms started. Imaging showed diffuse bilateral pulmonary infiltrates rather than segmental or lobar infiltrates especially in dependent pulmonary segments.
Transfusion-related acute lung injury	Sudden onset of symptoms	No blood products were transfused before the symptoms started.
Amniotic fluid embolism	The symptoms started after delivery. Rapid onset and worsening of symptoms are typical for amniotic fluid embolism. One-fourth of the cases of amniotic fluid embolism present with an atypical presentation, which may lack the other clinical features. No fever was present during labor.	Amniotic fluid embolism is a rare cause of ARDS. The symptoms usually start within 30 minutes of delivery. No hemodynamic instability, cardiovascular collapse, seizures, or DIC were observed.

Sepsis is the most common cause of ARDS, and it should be the first differential in consideration in patients with severe infection or new hypotension. Sepsis causes ARDS mainly secondary to systemic inflammation leading to increased pulmonary capillary endothelial permeability. Our patient had nonspecific symptoms related to sepsis, including fever, tachycardia, tachypnea, and leukocytosis, and she met the systemic inflammatory response syndrome (SIRS) criteria for sepsis (as shown in Table 2); however, most of these symptoms are nonspecific and can be seen in systemic inflammation from various etiologies. SIRS criteria are sensitive but lack specificity compared to qSOFA (Quick SOFA) criteria, and the patient met only one qSOFA criterion (RR > 22/min). Furthermore, she remained hemodynamically stable despite severe ARDS, and no definitive source of infection was found [6].

Infectious pneumonia is a common cause of ARDS outside the hospital and is mostly caused by COVID-19, *Streptococcus pneumonia*, *Legionella pneumophila*, and gram-negative bacilli. It is unlikely to be the cause of ARDS in our patient as infectious pneumonia presents with cough, dyspnea, and fever and is unlikely to cause sudden onset ARDS in a patient without any preceding respiratory symptoms [7,8]. Furthermore, laboratory workup including respiratory viral panel (includes COVID-19 PCR) and respiratory bacterial cultures was normal. Our patient was fully vaccinated against COVID-19.

Aspiration pneumonia is a recognized complication of general anesthesia, trauma, and ICU patients, especially with an altered level of consciousness. The gastric contents with acidic pH with or without particulate food are aspirated into the lungs, which leads to initial chemical pneumonitis and later may get infected and develop into bacterial pneumonia. Severity can range from mild pneumonitis to severe ARDS. Most events leading to aspiration are either unwitnessed or silent. Our patient did not have any risk factors for aspiration, and no aspiration events were observed. Aspiration can cause chemical pneumonitis initially, however, unlikely to cause a sudden onset of severe ARDS with dense bilateral infiltrates as initial presentation [9].

Transfusion-related acute lung injury (TRALI) is a clinical diagnosis and develops during or shortly after blood product transfusion is performed. ARDS should develop within six hours of blood product transfusion to meet the diagnostic criteria for TRALI. Our patient did not receive any blood transfusions before or during the symptoms started, making this diagnosis unlikely [10].

AFE remains one of the most dreadful complications of pregnancy. It is a rare condition, and the incidence varies in different parts of the world, usually between 1.9 and 6.1 cases per 100,000 deliveries [11]. It was initially reported in 1926, though it was described in the case series for the first time in 1941. The pathogenesis is not clear; however, it is believed to result from the entry of amniotic fluid into the maternal systemic circulation secondary to disruption of a barrier between the amniotic fluid and maternal circulation usually at the time of delivery. Entry of amniotic fluid into maternal circulation results in a severe inflammatory response that causes clinical manifestations of AFE. There is no mechanical obstruction of pulmonary vasculature from the amniotic fluid; however, elevated pulmonary pressures are still seen due to ARDS.

Elevated pulmonary pressures and the direct effect of the inflammatory mediators on the myocardium may lead to cardiac dysfunction, which may also contribute to pulmonary edema. Symptoms usually start during labor or 30 minutes after delivery; however, cases occurring during the first and second trimesters have been reported. Onset is abrupt most of the time, and the symptoms progress rapidly in a catastrophic manner. Clinical features include hypoxic respiratory failure secondary to ARDS, hemodynamic compromise usually from cardiac arrest (typically ventricular tachycardia or fibrillation), and bleeding secondary to disseminated intravascular coagulopathy (DIC). Laboratory workup may show findings suggestive of DIC (elevated D-dimer, low fibrinogen, and thrombocytopenia), leukocytosis, and anemia (secondary to hemorrhage). Chest imaging shows dense bilateral infiltrates. The diagnosis is clinical, and it is a diagnosis of exclusion as there is no confirmatory test available [1,12]. However, all clinical findings are not seen in every patient, and there are many atypical cases described in case reports. One-fourth of all patients present only with hypotension and acute respiratory failure. Rarely, DIC may be an initial presentation, or it may be absent [13]. Our patient may have atypical AFE as the patient was completely asymptomatic at the time of admission, and the symptoms rapidly developed and progressed after vaginal delivery to the point that she required intubation and later ECMO over less than 24 hours. Sepsis can be another possibility; however, AFE is more likely to cause a rapid onset of worsening ARDS after delivery in a patient without any evidence of sepsis or septic shock.

Other risk factors for ARDS include lung and hematopoietic stem cell transplant, drug overdose and idiosyncratic reaction, severe trauma including lung contusion and fat embolism, acute pancreatitis, near drowning, thoracic surgery, etc., none of which were observed or suspected in our patient.

## Conclusions

ARDS is characterized by noncardiogenic pulmonary edema and is one of the most common diagnoses requiring ICU admission. It presents with acute onset hypoxic respiratory failure, associated with new bilateral pulmonary infiltrates. It is important to promptly diagnose and treat ARDS to reduce the associated high mortality. There are over 60 known causes of ARDS, and the list is getting longer with time. AFE is a very rare cause of ARDS, which is seen within six hours after delivery, and is usually associated with hemodynamic instability secondary to cardiac arrhythmias and bleeding secondary to DIC. Many atypical cases of AFE have been reported. Our patient did not have a clear cause of ARDS, and after ruling out the other diagnoses, the atypical AFE remained the most likely explanation for ARDS. The purpose of this case report is to highlight the importance of having atypical AFE among the differential diagnoses if ARDS develops in a pregnant woman especially after delivery, even if some of the clinical features of AFE are missing. 
